# Changes of CD90 expression and immunoreactive cell localisation in rat dental pulp after cavity preparation

**DOI:** 10.1111/aej.12307

**Published:** 2018-09-21

**Authors:** Yousuke Sano, Akina Sugiuchi, Keisuke Mitomo, Akihide Yanagisawa, Ryo Kambe, Masahiro Furusawa, Takashi Muramatsu

**Affiliations:** ^1^ Department of Endodontics Tokyo Dental College Chiyoda‐Ku, Tokyo Japan; ^2^ Department of Operative Dentistry, Cariology and Pulp Biology Tokyo Dental College Chiyoda‐Ku, Tokyo Japan

**Keywords:** cavity preparation, CD90, dental pulp, regeneration, subodontoblastic layer

## Abstract

CD90 expression and immunoreactive cell localisation in rat dental pulp cells after cavity preparation was investigated. Cavity preparation was performed on the maxillary first molar of 8‐week‐old Wistar rats (*n* = 36), and immunohistochemistry and quantitative real‐time PCR were performed. CD90‐immunoreactivity was observed among subodontoblastic cells in the control group. One day after cavity preparation, the CD90‐immunoreactivity disappeared under the cavity area. While CD90‐immunoreactivity was faint after 3 days, the re‐arrangement of odontoblasts was detected in contact with dentine. After 5 days, the odontoblasts were observed beneath the dentine, and CD90‐immunoreactive cells were localised under the odontoblast layer. Immunofluorescence showed co‐localisation of CD90 and nestin was detected after 3 days. After 5 days, CD90‐immunoreactivity increased at the subodontoblastic layer. mRNA expression of CD90 and DSPP decreased after cavity preparation, and gradually recovered (*P* < 0.01). These results suggest that CD90‐immunoreactive cells in the subodontoblastic layer contribute to regeneration of odontoblast and subodontoblastic layers following cavity preparation.

## Introduction

Various stimuli, including dental caries and mechanical, physical and chemical injuries, have been suggested to irritate the dentine/pulp complex. The removal of infected dentine and cavity preparation are commonly performed in clinical settings. Cavity preparation induces degenerative changes, acute inflammation and formation of tertiary dentine in dental pulp at the affected site 1, 2, 3, 4, 5, 6, 7, 8. However, the mechanisms underlying the regeneration of dental pulp tissue after cavity preparation have not yet been elucidated in detail.

Stem cells play an important role for regeneration, and have two major properties: self‐renewal and differentiation. These cells have the potential to differentiate into various cells such as adipogenic, cementogenic, neurogenic, osteogenic and chondrogenic cells 9. Mesenchymal stem cells (MSCs) exist in various tissues and may differentiate into osteoblasts, adipocytes and chondrocytes *in vitro*. Since 2000, MSCs have been isolated from the dental pulp of permanent teeth, and are generally referred to as dental pulp stem cells (DPSCs) 10. Stem cells were subsequently isolated from deciduous teeth, and are referred to as stem cells from exfoliated deciduous teeth (SHED) 11. DPSCs and SHED both show multi‐potency with differentiation into osteoblasts, chondrocytes, adipocytes and neuronal cells as well as bone marrow mesenchymal stromal cells. DPSC were initially isolated by growth potential, and the Mesenchymal and Tissue Stem Cell Committee of the International Society for Cellular Therapy proposed minimal criteria to define human MSC in a position paper 12. The expression of cell surface antigen markers is important; MSC must express CD105, CD73 and CD90, but not CD45, CD34, CD14 or CD11b, CD79 alpha or CD19 and HLA‐DR surface molecules 12. Since the publication of this position paper, DPSC and SHED have been isolated using various cell surface markers 13; however, the localisation of DPSC remains unknown.

CD90, one of the MSC surface markers, was originally a T‐cell marker and expressed in thymocytes. Since CD90 was identified as an MSC marker, it has been attracting attention in regenerative research 14, 15. The expression of CD90 has been reported in DPSC 10, 11, 16, 17, and a recent study demonstrated that CD90‐immunoreactive cells localised in the subodontoblastic layer of dental pulp 18. However, the role of CD90‐immunoreactive cells during the regenerative process after cavity preparation has not yet been clarified.

In this study, the localisation of CD90‐immunoreactive cells was investigated in dental pulp tissue after cavity preparation, and whether CD90‐immunoreactive cells contribute to the regeneration of rat dental pulp tissue was examined.

## Materials and methods

All experiments were performed according to the Guidelines for the Treatment of Animals at Tokyo Dental College (Approval No.272101). Thirty‐six male Wistar rats weighing approximately 250 g were used in this study.

### Cavity preparation

Rats were deeply anaesthetised by the inhalation of isoflurane (Wako Pure Chemical Industries, Ltd., Tokyo, Japan) and an intraperitoneal injection of sodium pentobarbital (Somnopentyl® Kyoritsuseiyaku Co., Tokyo, Japan) (0.1 mL/100 g) immediately before use. Cavity preparation was performed as described previously 6. In brief, a groove‐shaped cavity was prepared on the mesial surface of the upper right first molar with a straight hand piece and tungsten carbide bur (1 mm in diameter, Dentsply‐Maillefer, Ballaigues, Switzerland) under water‐cooling. The upper left first molar of the same rat was used as a control.

### Tissue preparation

Rats were sacrificed 1, 3 and 5 days after cavity preparation. At each time point, the perfusion fixation of 4% paraformaldehyde ‐ 0.1 M phosphate buffer (PFA; pH7.4) was performed transcardially, and dissected upper jaws were immersed for 24 h in the same fixative. Tissues were then decalcified for 2 weeks in 10% ethylenediaminetetraacetic acid (EDTA) containing 7% sucrose. Tissues were embedded in paraffin and then cut to obtain 4‐μm‐thick sagittal sections for immunohistochemistry. Regarding immunofluorescence, PFA‐fixed tissues were decalcified in 10% EDTA containing 7% sucrose. After decalcification, 8‐μm‐thick frozen sections were prepared.

### Immunohistochemistry

**Figure 1:**
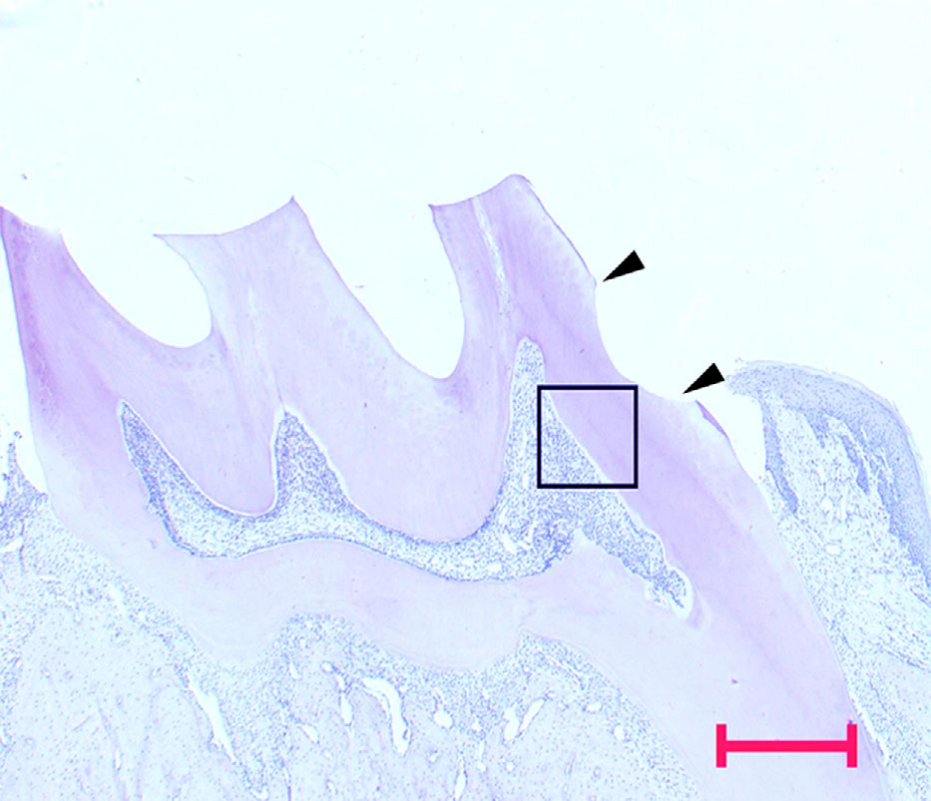
Observation area used in this study: The mesial surface of the upper right first molar in a Wistar rat is prepared. Arrowheads indicate the drilled area, and the histological observation area is framed in the specimen. The Figure is a specimen of 0 day after cavity preparation, Bar = 500 μm.

The streptavidin–biotin immunoperoxidase method was employed for immunohistochemistry using the Histofine simple stain rat MAX‐PO (MULTI; Nichirei Bioscience Co., Ltd., Tokyo, Japan). Sections were deparaffinised with xylene, washed with graded ethanol, and then washed again with distilled water. Sections were microwaved at 65°C for 20 min in 0.01 M citrate buffer (pH 6.0), cooled to room temperature (RT) and washed in distilled water. Endogenous peroxidase activity was blocked by incubating the sections with 0.3% H_2_O_2_ in methanol for 30 min and then washing in phosphate‐buffered saline (PBS, pH7.2, 0.01M) three times for 5 min each. In order to prevent non‐specific reactions, sections were incubated with 10% goat serum for 30 min. Anti‐rat CD90 (Thy‐1; dilution 1:100, eBioscience, CA, USA) mouse antibody was incubated at RT for 1 h. Horseradish peroxidase (HRP)‐conjugated anti‐mouse IgG was then incubated at RT for 30 min. After washing in PBS three times for 5 min each, 3,3′‐diaminobenzidine‐tetrahydrochloride (DAB) in 0.05 M Tris–HCl buffer (pH7.6) was used to visualise reactivity. After washing in distilled water, sections were counter‐stained with haematoxylin for 30 s and then observed under a light microscope (Axiophot2; Carl Zeiss, Oberkochen, Germany). Drilled dentine of the upper right first molar and the observation area were shown in Fig. 1.

### Immunofluorescence

The frozen sections were then blocked with 10% normal goat serum at RT for 30 min, and incubated with FITC‐conjugated anti‐rat CD90 mouse monoclonal antibody (1:100, HIS51, eBioscience) and anti‐rat nestin (1:100, Santa Cruz Biotechnology, CA, USA) rabbit antibody at RT for 1 h. After washing in PBS, sections were incubated at RT for 30 min with AlexaFluor^®^568‐conjugated ant‐rabbit antibody (1:200, Molecular Probes, Eugene, OR, USA) for nestin. After washing in PBS three times for 5 min each, specimens were sealed with a liquid mounting agent (Prolong Gold Antifade Mountant with DAPI, Life Technologies Inc., Carlsbad, CA, USA) and observed under a confocal laser‐scanning microscope (LSM5 DUO; Carl Zeiss).

### RNA extraction and quantitative real‐time PCR

Rats were sacrificed 1, 3 and 5 days after cavity preparation. Regarding RNA extraction, teeth were extracted with an explorer and coronal and mesial root pulp tissues were collected. Total RNA was extracted from pulp by a modified acid–guanidinium–thiocyanate–phenol–chloroform (AGPC) method using a Teflon‐glass homogeniser and TRIzol^®^ (Invitrogen, Carlsbad, CA, USA) according to the manufacturer's protocol. The quantitation of RNA was performed with NanoDrop (ND‐2000; Thermo Fisher Scientific, MA, USA). RNA was reverse‐transcribed into cDNA using TaqMan Reverse Transcription Regents (Applied Biosystems, Foster City, CA, USA). In each tissue sample, 1 μg of total RNA was reverse‐transcribed using a random primer, and the products obtained were subjected to PCR amplification under the same conditions as described above. The reaction mixture was added to the RNA solution, pre‐incubated at 25°C for 10 min, incubated at 48°C for 30 min, heated at 94°C for 5 min and chilled at 4°C. qRT‐PCR was performed using TaqMan MGB probes (Applied Biosystems), the TaqMan Fast Universal PCR Master Mix (Applide Biosystems), and 7500 Fast Real‐Time PCR System (denature 95°C, 3 s; annealing/extension 60°C, 30 s, 40 cycles, Applied Biosystems). The TaqMan MGB probes and primer sets for the rat *CD90* (*Thy‐1*, Rn00562048_m1) and *dentine sialophosphoprotein* (*DSPP*, Rn02132391_s1) genes and *18S*, as an endogenous control, were purchased from Applied Biosystems. Quantification was performed using ABI 7500 fast system software (Applied Biosystems) and compared with the ΔΔCt method. Experiments were repeated in triplicate.

### Statistical analysis

Data were expressed as the mean ± SD. The significance of differences between groups was evaluated by performing one‐way analysis of variance (anova) and the Dunnet test. Differences at *P* < 0.01 were considered to be significant.

## Results

### Immunohistochemistry

**Figure 2:**
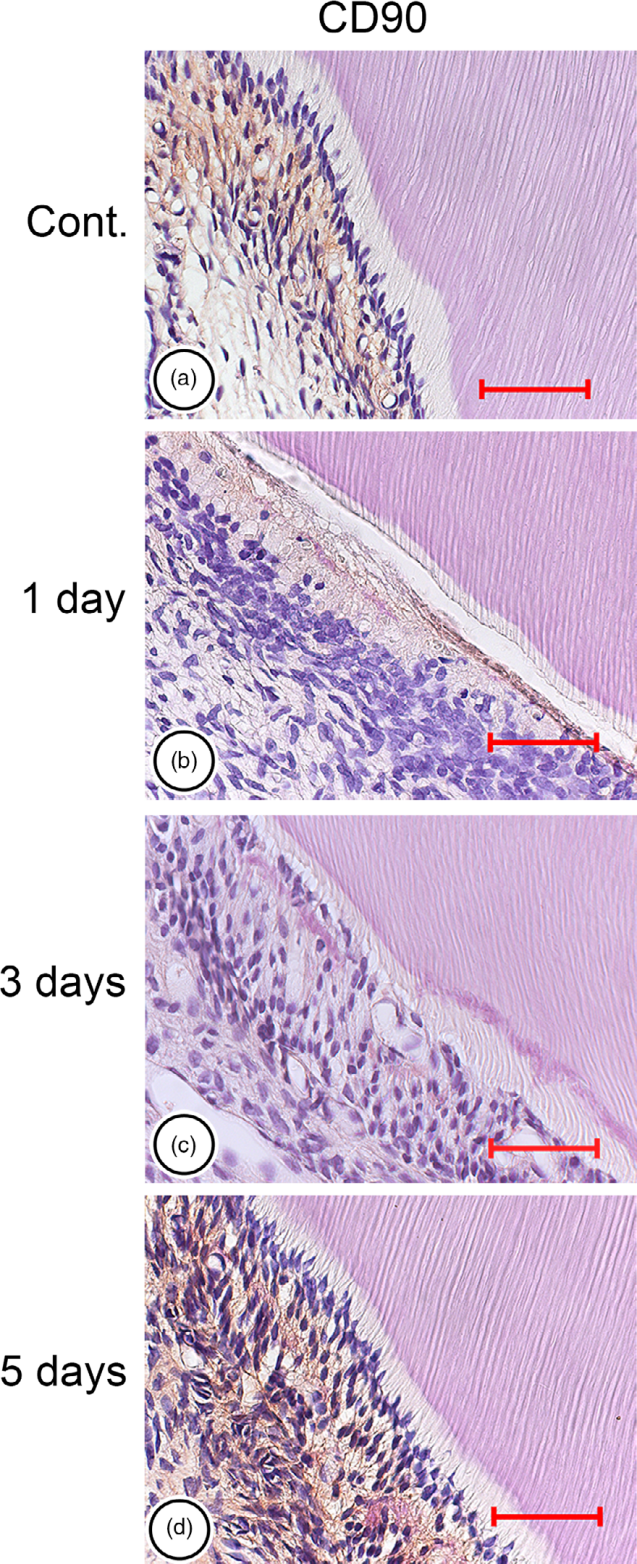
Immunohistochemistry. In the control group, CD90‐immunoreactive cells were mainly observed at the subodontoblastic layer of coronal pulp (a). One day after preparation, the immunoreactivity of CD90 disappeared just under the cavity area (b). After 3 days, CD90‐immunoreactive cells were not present, whereas the rearrangement of cells indicating odontoblasts was detected in contact with the dentine (c). After 5 days, CD90‐immunoreactive cells localised at the subodontoblastic layer again (d). Bars = 50 μm.

We investigated the odontoblast layer beneath the dentine and subodontoblastic layer as shown in box of Fig. 1. In the control group, CD90‐immunoreactive cells were mainly observed in the subodontoblastic layer of coronal pulp (Fig. 2a). Immunoreactivity was not detected at odontoblasts lining the predentine and at pulp cells in the central region of the pulp. One day after preparation, odontoblasts were detached from predentine and odontoblast area became oedematous. The immunoreactivity of CD90 disappeared just under the cavity area (Fig. 2b). After 3 days, the rearrangement of cells resembling odontoblasts was seen in contact with the dentine, and the CD90‐immunoreactive cells were faintly detected (Fig. 2c). Capillary was also seen in the rearrangement cells. After 5 days, CD90‐immunoreactive cells appeared again mainly in the subodontoblastic layer (Fig. 2d).

### Immunofluorescence

**Figure 3:**
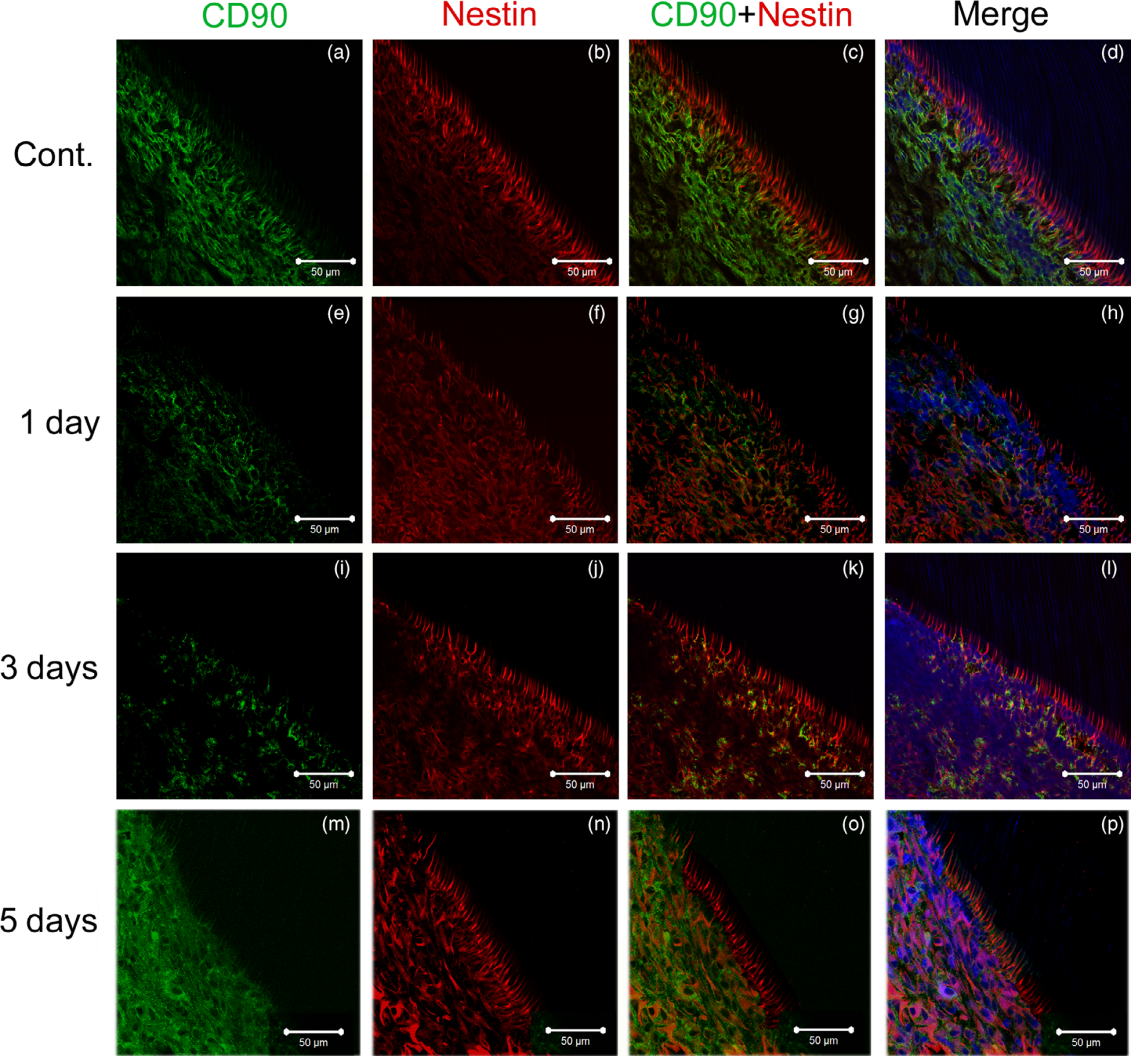
Immunofluorescence. In the control group, CD90‐immunoreactive cells (green) were mainly observed at the subodontoblastic layer (a). The immunoreactivity of nestin (red) was detected in the odontoblast layer and dentinal fibres (b). The co‐localisation of CD90 and nestin was not defined (c, d). One day after preparation, the immunoreactivity of CD90 decreased and its expression was mottled (e). The expression of nestin decreased in the odontoblast layer and dentinal tubules and was observed in the cell‐rich layer (f). Double‐immunoreactive cells for CD90 and nestin were not seen (g, h). After 3 days, the expression of CD90 became faint and scanty, whereas the immunoreactivity of nestin was unchanged, except for a decrease in dentinal fibres (i, j). Double‐immunoreactive cells for CD90 and nestin were yellow in colour and were noted at the superficial layer of pulp and odontoblasts (k, l). After 5 days, the immunoreactivity of CD90 was noted again at the regenerative subodontoblastic layer. Furthermore, blood vessels were noted in pulp (m). The immunoreactivity of nestin was detected in spindle‐shaped cells in pulp (n). In merged figures, the co‐localisation of CD90 and nestin was noted in spindle‐shape cells in the subodontoblastic layer (o, p). Green: CD90, Red: nestin, Blue: nuclear (stained with DAPI), Bars = 50 μm.

To investigate differentiation into odontoblasts, immunofluorescence for CD90 and nestin was carried out. In the control group, CD90‐immunoreactive cells were mainly observed in the subodontoblastic layer (Fig. 3a). The immunoreactivity of nestin (odontoblast marker) was detected in the odontoblast layer and dentinal fibres (Fig. 3b). The co‐localisation of CD90 and nestin was not defined (Fig. 3c,d). One day after preparation, the immunoreactivity of CD90 decreased and its expression was mottled (Fig. 3e). The expression of nestin decreased in the odontoblast layer and dentinal tubules, and was also observed in the cell‐rich layer (Fig. 3f). Double‐immunoreactive cells for CD90 and nestin were not seen (Fig. 3g,h). After 3 days, the expression of CD90 became faint and scanty, whereas the immunoreactivity of nestin was unchanged, except for a decrease in dentinal fibres (Fig. 3i,j). Double‐immunoreactive cells for CD90 and nestin were yellow in colour and were noted at the superficial layer of pulp and odontoblasts (Fig. 3k,l). After 5 days, the immunoreactivity of CD90 was observed again at the regenerative subodontoblastic layer (Fig. 3m). The immunoreactivity of nestin was detected in odontoblast layers and spindle‐shaped cells in pulp (Fig. 3n). In merged figures, the co‐localisation of CD90 and nestin was not observed in odontoblast layer and subodontoblastic layer (Fig. 3o,p).

#### Expression of mRNA

**Figure 4:**
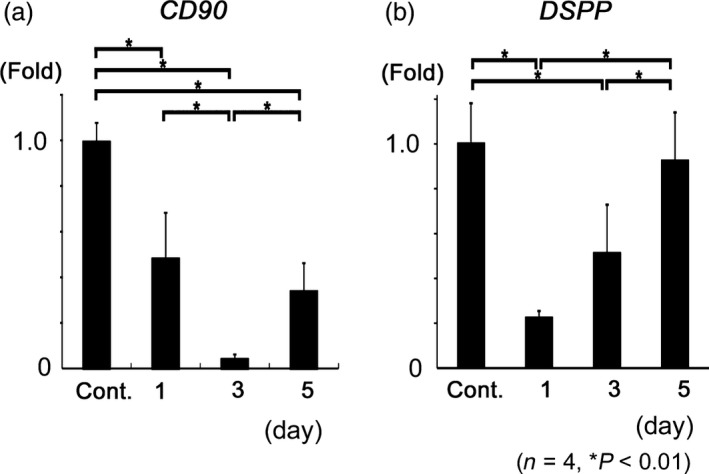
mRNA expression. (a) The expression of *CD90* significantly decreased 1 day after cavity preparation (*P* < 0.01), and gradually decreased by 3 days (*P* < 0.01). After 5 days, the expression of *CD90* increased (*P* < 0.01) to similar levels as those observed 1 day after cavity preparation. (b) *DSPP* expression levels significantly decreased to approximately 25% of those observed 1 day after preparation (*P* < 0.01). The expression of *DSPP* gradually increased and reached control levels after 5 days. Data were expressed as the mean ± SD.

The expression of *CD90* markedly decreased 1 day after preparation (*P* < 0.01), and gradually decreased by 3 days (*P* < 0.01). After 5 days, the expression of *CD90* increased (*P* < 0.01) to similar levels as those on 1 day (Fig. 4a). The expression of *DSPP* markedly decreased to approximately 25% of that observed 1 day after preparation (*P* < 0.01). The expression of *DSPP* gradually increased and, after 5 days, reached control levels (Fig. 4b).

## Discussion

### Changes in dental pulp tissue after cavity preparation

Changes in dental pulp after cavity preparation have been investigated histologically, and typical findings are the aspiration of odontoblasts into dentinal tubules and inflammatory cell infiltration in dental pulp 3, 19, 20. Severe inflammation injures pulpal cells, reduces pulpal vitality and ultimately results in the necrosis of all tooth pulp 21. Previous studies reported that cavity preparation induced the degeneration at odontoblasts and deeper pulpal cells 22, 23. Dental pulp regeneration after degenerative changes was previously shown to be completed by the proliferation and differentiation of progenitor cells and mesenchymal cells in the subodontoblastic layer 6, 22, 24, 25. However, role of CD90‐positive cells has not been investigated. The disarrangement and disappearance of odontoblasts and subodontoblastic cells were observed in this study. Furthermore, CD90 decreased 1 day after cavity preparation and then increased by 5 days in immunohistochemical staining and qRT‐PCR. These results imply that odontoblasts and subodontoblastic cells degenerate after cavity preparation, and CD90‐immunoreactive cells are associated with regeneration of odontoblasts and subodontoblastic cells.

### Differentiation after cavity preparation, and its relationship with stem cells

Osteogenicities and therapeutic potentials of DPSC and SHED for bone regeneration and use in regenerative medicine have been investigated, since their isolation from dental pulp. However, limited information is currently available on the role of stem cells after cavity preparation in situ. Furthermore, the types of cells that differentiate into odontoblasts remain unclear. An earlier in vivo study showed that daily intraperitoneal injections of bromodeoxyuridine (BrdU) into pregnant rats successfully labelled newly odontoblast‐like cells, and the co‐expression of stem cell markers such as STRO‐1 and CD146 was observed after cavity preparation 26. However, CD90 has never been investigated in dental pulp after cavity preparation even though it was proposed to be an MSC marker. In this study, we employed CD90, a stem cell marker in normal dental pulp 18, 27. An immunofluorescent study showed that CD90 and nestin were sporadically co‐localised at the odontoblast and subodontoblastic layers 3 day after cavity preparation. These results suggest that some CD90‐immunoreactive cells differentiate into odontoblasts after cavity preparation. Most CD90‐immunoreactive cells did not express nestin 5 days after cavity preparation. Previous studies demonstrated that CD90‐highly immunoreactive cells had the ability to differentiate into odontoblasts 18, 27. Two types of CD90‐immunoreactive cells appear to exist: one that differentiates into odontoblasts and another that regenerates the subodontoblastic layer in dental pulp.

### Regeneration of the subodontoblastic area by CD90‐immunoreactive cells

In this study, CD90‐immunoreactive cells localised at the subodontoblastic layer again after 5 days, suggesting regeneration of the subodontoblastic layer. Interestingly, nestin‐immunoreactive cells were also detected at the subodontoblastic layer after 5 days in this study. Bone marrow MSC localised adjacent to vessels and were identified by typical mesenchymal markers (CD73, CD90 and CD105) as well as CD146 and nestin 28. Furthermore, nestin was sporadically expressed in mature endothelial cells and was consistently expressed in adult angiogenic vasculature. Nestin‐positive cells were reported to participate in angiogenesis as MSCs or endothelial progenitor cells (EPCs) in several tissues 29. The results of this study, taken together with previous findings, imply that CD90 and nestin are associated with regeneration of the subodontoblastic layer in cooperation.

In conclusion, we herein showed changes in the localisation of CD90‐immunoreactive cells in rat dental pulp after cavity preparation, and CD90‐immunoreactive cells appear to differentiate into odontoblastic cells and contribute to regeneration of the subodontoblastic layer after cavity preparation.

## Authorship declaration

All authors have contributed significantly and agreed with the manuscript.

## Disclosure statement

The authors deny any conflicts of interest related to this study.
